# Construction of a competitive endogenous RNA network and analysis of potential regulatory axis targets in glioblastoma

**DOI:** 10.1186/s12935-021-01789-z

**Published:** 2021-02-12

**Authors:** Kai Yu, Huan Yang, Qiao-li Lv, Li-chong Wang, Zi-long Tan, Zhe Zhang, Yu-long Ji, Qian-xia Lin, Jun-jun Chen, Wei He, Zhen Chen, Xiao-li Shen

**Affiliations:** 1 Department of Neurosurgery, The Second Affiliated Hospital of Nanchang University, No. 1 Minde Road, Donghu District, Jiangxi 330006 Nanchang, People’s Republic of China; 2 Jiangxi Key Laboratory of Translational Cancer Research, Jiangxi Cancer Hospital, Jiangxi Nanchang, People’s Republic of China; 3 Jiangxi University of Traditional Chinese Medicine, Jiangxi Nanchang, People’s Republic of China

**Keywords:** Glioblastoma, Bioinformatics analysis, Competitive endogenous RNA, Differentially expressed gene

## Abstract

**Background:**

Glioblastoma is the most common primary malignant brain tumor. Because of the limited understanding of its pathogenesis, the prognosis of glioblastoma remains poor. This study was conducted to explore potential competing endogenous RNA (ceRNA) network chains and biomarkers in glioblastoma by performing integrated bioinformatics analysis.

**Methods:**

Transcriptome expression data from The Cancer Genome Atlas database and Gene Expression Omnibus were analyzed to identify differentially expressed genes between glioblastoma and normal tissues. Biological pathways potentially associated with the differentially expressed genes were explored by Gene Ontology and Kyoto Encyclopedia of Genes and Genomes pathway analysis, and a protein-protein interaction network was established using the STRING database and Cytoscape. Survival analysis using Gene Expression Profiling Interactive Analysis was based on the Kaplan–Meier curve method. A ceRNA network chain was established using the intersection method to align data from four databases (miRTarBase, miRcode, TargetScan, and lncBace2.0), and expression differences and correlations were verified by quantitative reverse-transcription polymerase chain reaction analysis and by determining the Pearson correlation coefficient. Additionally, an MTS assay and the wound-healing and transwell assays were performed to evaluate the effects of complement C1s (C1S) on the viability and migration and invasion abilities of glioblastoma cells, respectively.

**Results:**

We detected 2842 differentially expressed (DE) mRNAs, 2577 DE long non-coding RNAs (lncRNAs), and 309 DE microRNAs (miRNAs) that were dysregulated in glioblastoma. The final ceRNA network consisted of six specific lncRNAs, four miRNAs, and four mRNAs. Among them, four DE mRNAs and one DE lncRNA were correlated with overall survival (*p* < 0.05). C1S was significantly correlated with overall survival (*p*= 0.015). In functional assays, knockdown of C1S inhibited the proliferation and invasion of glioblastoma cell lines.

**Conclusions:**

We established four ceRNA networks that may influence the occurrence and development of glioblastoma. Among them, the MIR155HG/has-miR-129-5p/C1S axis is a potential marker and therapeutic target for glioblastoma. Knockdown of C1S inhibited the proliferation, migration, and invasion of glioblastoma cells. These findings clarify the role of the ceRNA regulatory network in glioblastoma and provide a foundation for further research.

## Background

Glioblastoma (GBM) is a common malignant primary brain tumor, accounting for approximately 57 % of all gliomas and 48 % of all primary malignant central nervous system tumors[1]. The three standard modes of treatment (maximal surgical resection, radiotherapy, and chemotherapy) result in an average survival of only 14 months after diagnosis[2, 3]; without treatment, the life expectancy is less than 6 months[4]. The pathogenesis, development, reproduction, and molecular mechanisms of GBM are not well-understood; this hinders the research and development of precise treatments for this condition[5]. Therefore, studies of the molecular mechanisms underlying the action of GBM and new therapeutic strategies are urgently needed.

High-throughput sequencing technologies are powerful tools for identifying potential biomarkers useful for the diagnosis and treatment of cancer. Integrating bioinformatics knowledge with transcriptome sequencing can make data analysis and differential gene identification faster and more accurate[6, 7]. Consequently, studies aimed at applying bioinformatics strategies to analyze molecular markers and diagnostic indicators of GBM are urgently needed.

The competing endogenous RNA (ceRNA) hypothesis proposed in 2011 described an intricate post-transcriptional regulatory network that mainly includes long non-coding RNAs (lncRNAs), microRNAs (miRNAs), mRNAs, circular RNAs (circRNAs), and other types of RNAs[8]. Recent studies have focused on roles of ceRNAs in the tumorigenesis and development of various cancers[9, 10], including lung cancer[10], gallbladder cancer[10], glioblastoma[11], gastric cancer[12], pancreatic cancer[13], and colorectal cancer[14]. The molecular mechanisms underlying the role of the ceRNA regulatory network in the oncogenesis and development of various cancers have also been examined[15, 16]. However, to date, few ceRNA networks for GBM have been constructed using bioinformatics analysis.

To improve the understanding of ceRNA-based regulatory mechanisms in GBM, we constructed an aberrant lncRNA-miRNA-mRNA network using a comprehensive bioinformatics approach. The expression of crucial RNAs in clinical specimens was analyzed by real-time quantitative reverse-transcription polymerase chain reaction (qRT-PCR). Additionally, the biological functions of crucial RNAs were evaluated in vitro. Our study may facilitate comprehensive analysis of essential molecules, aid in the identification of novel mechanisms underlying glioblastoma pathogenesis, and reveal potential therapeutic targets for treating GBM.

## Methods

### Microarray data

Three expression profile datasets [GSE90604 (GPL17692 platform), GSE65626 (GPL17586 platform), and GSE116520 (GPL10558 platform)] and two microRNA expression profile datasets [GSE65626 (GPL19117 platform) and GSE90604 (GPL21572 platform)] were downloaded from the National Center of Biotechnology Information Gene Expression Omnibus (GEO) (https://www.ncbi.nlm.nih.gov/geo/) database. The lncRNA transcriptome data for GBM were downloaded from The Cancer Genome Atlas (TCGA; https://cancergenome.nih.gov/) database; these data included 5 cases of para-cancerous tissues and 156 cases of tumor tissues. The GSE90604 dataset included 16 GBM tissue samples and 7 healthy brain tissue samples; GSE65626 included 6 GBM and 6 healthy brain tissue samples; and GSE116520 included 17 GBM tumor core tissue samples, 17 GBM peritumoral brain zone tissue samples, and 17 healthy brain tissue samples. Two GEO datasets (GSE65626, GSE90604) with miRNA and mRNA simultaneously detected by Affymetrix microarray were included to reduce variability between samples. Data were normalized using limma software and the edgeR package[17].

### Identification of differentially expressed genes (DEGs)

The three expression profiling datasets used for analysis included CEL format files. These datasets of differential RNA expression in GBM were compared with data from normal brain tissue using the limma software package in R. To establish statistical significance, the GSE90604 (miRNA and mRNA) and TCGA datasets were filtered using false discovery rate (FDR) < 0.01 and |fold-change (FC)| > 1 as cutoffs. GSE65626 (miRNA and mRNA) was filtered using cutoffs of p < 0.05 and |FC| > 1. GSE116520 (mRNA) was filtered using cutoffs of FDR < 0.01 and |FC| > 1.5. A Venn diagram was constructed using Venny 2.1.

### Gene ontology (GO) and pathway enrichment analysis

GO is widely used in bioinformatics to mainly study three aspects of biology: biological processes (BP), molecular functions (MF), and cellular components (CC). The Kyoto Encyclopedia of Genes and Genomes (KEGG) reflects biological molecular interactions and chemical reactions. We used the Database for Annotation, Visualization, and Integrated Discovery (DAVID) to analyze the DEGs. Values of *p*< 0.05 and gene counts ≥ 8 were considered as statistically significant.

### Protein‐protein interaction (PPI) network and gene module analysis

First, the Search Tool for the Retrieval of Interacting Genes (STRING) database was used to obtain DEG-encoded protein and PPI information. Second, PPI pairs with a combined score > 0.4 were downloaded and analyzed. The PPI networks were constructed using Cytoscape software. The plug-in Molecular Complex Detection (MCODE) was used to screen modules or clusters in large PPI networks. The following parameters were used for DEG clustering and scoring: MCODE score > 5, degree cutoff = 2, node score cutoff = 0.2, max depth = 100, and k-score = 2.

### Survival analysis of DEGs

Gene Expression Profiling Interactive Analysis (GEPIA), a web server for cancer and normal gene expression profiling and interactive analysis, was used to perform survival analysis of the DEGs, confirm their expression, and identify the median expression among tumor and normal samples in BodyMap[18].

### Prediction of target lncRNA-miRNA genes and initial lncRNA-miRNA-mRNA network construction

The initial lncRNA-miRNA-mRNA network was constructed according to the ceRNA hypothesis. miRcode, the miRTarBase database, and TargetScan were used to predict target differentially expressed (DE) mRNAs, and only miRNA-mRNAs that were aligned across the three databases were incorporated into the ceRNA network. Next, DE lncRNA-DE miRNA interactions were predicted based on the miRcode database. Cytoscape (Version 3.7.1) was used to construct the preliminary ceRNA network.

### Gene expression and final ceRNA network construction

**Fig. 1:**
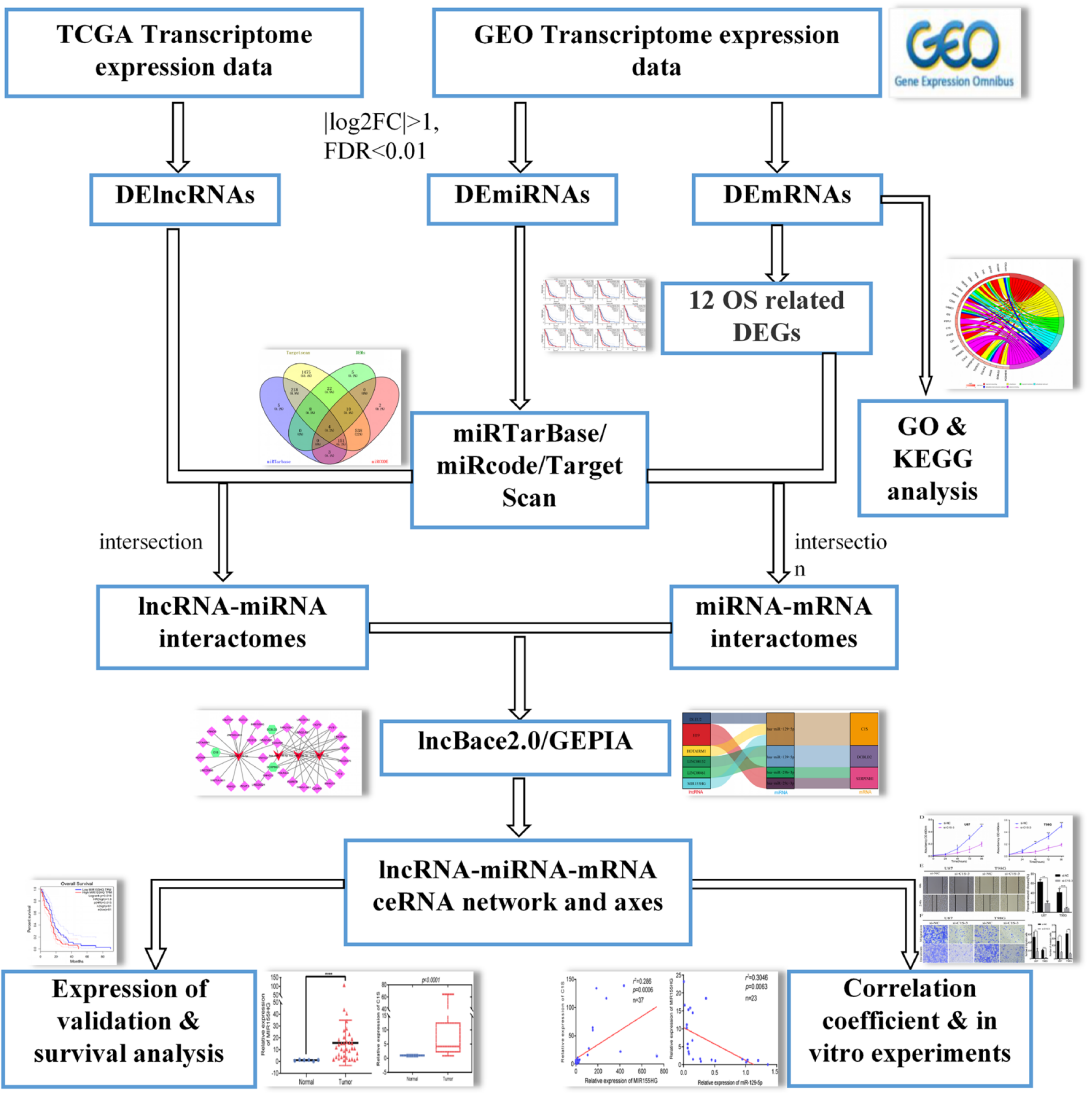
Flow chart of ceRNA network and control axis construction in glioblastoma

One-way analysis of variance was used to analyze the DEGs and DE lncRNAs. The cutoffs for the gene expression boxplot were set to |Log2FC| = 1 and p = 0.01. lncBace2.0 and GEPIA (http://gepia.cancer-pku.cn/index.html) were used to validate the DE lncRNAs and screen for genes showing significant expression, respectively. A Ji mulberry was used to construct the final lncRNA-miRNA-mRNA network. The flowchart for the simplified procedure is shown in Fig. 1.

### Patient samples

The 37 primary glioma samples were obtained from patients who underwent surgical treatment at the Xiangya Hospital of Central South University from September 2016 to May 2017 but were not administered chemotherapy or radiotherapy. Six normal brain tissues from patients with cerebral trauma surgery were collected as controls. All samples were snap frozen in liquid nitrogen until total RNA was extracted. This study was approved by the Central South University Xiangya Hospital Medical Ethics Committee (No. 201,803,806).

### Cell culture

The U87 and T98G cell lines were obtained from the American Type Culture Collection (Manassas, VA, USA). The cells were cultured in Dulbecco’s modified Eagle’s medium (HyClone, Logan, UT, USA) growth medium supplemented with 10 % fetal bovine serum (Gibco, Grand Island, NY, USA) at 37 °C in a humidified 5 % CO_2_ incubator.

### Cell transfection

The U87 and T98G cells were seeded into six-well plates and cultured until they reached 50–60 % confluence. They were then transiently transfected with small-interfering RNA (siRNA-*C1S*) and negative control (NC) siRNA, which were designed by RiboBio (Guangzhou, China), using Lipofectamine® RNAiMAX Transfection Reagent (Invitrogen, Carlsbad, CA, USA) according to the manufacturer’s instructions. This transfection reagent and siRNA were diluted in Opti-MEM (Invitrogen) without antibiotics and used for experiments at 48 h after transfection.

### RNA isolation and qRT-PCR

Total RNA was extracted from tissues using TRIzol reagent (TaKaRa, Shiga, Japan) according to the manufacturer’s protocol. cDNA was transcribed using the PrimeScript RT Reagent Kit (TaKaRa, RR047A). A SYBR Green PCR Kit (Takara, RR820A) was used to measure the quantity of isolated RNA according to the manufacturer’s instructions. qRT-PCR was performed using the CFX Connect Real-Time System (Bio-Rad, Hercules, CA, USA) as follows: 95 °C for 3 min, and 40 cycles of 95 °C for 10 s and 60 °C for 30 s. GAPDH was used as an internal control. The 2^−ΔΔCt^ method was used to calculate the relative expression levels of the RNAs. The qRT-PCR primers included the bulge-loop RT primer and qPCR primers specific for has-miR-129-5p that were designed and synthesized by RiboBio. The primers for lncRNA *MIR155HG* were F: 5′-CCACCCAATGGAGATGGCTCT A-3′; and R: 5′- GCAAAAACCCCTATCACGATTA-3′. The primers for mRNA *C1S* were F: 5′- TCCAAGTCCCATACAACAAACTC-3′, and R: 5′-CAAACCCCGTAA AACGCTCT-3′. *GAPDH*: F: 5′-CCCATCACCATCTTCCAGGAG-3′, R: 5′-GTTGTCATGGATGACCTTGGC-3′.

### Cell proliferation assay

Cell viability was measured in an MTS assay (Cell Titer 96 aqueous one solution reagent, Promega, Madison, WI, USA) according to the manufacturer’s instructions. Briefly, at 24 h before the experiment, cells (2 × 10^3^ cells) were seeded into 96-well plates, divided into two groups (*NC*, si-*C1S*-3), and cultured for 0, 24, 48, 72, 96 h. At the specified time, 10 µL of CellTiter 96 Aqueous One Solution Reagent was added to each well and incubated at 37 °C for 30 min. The quantity of formazan product was determined by measuring the absorbance at 490 nm with a microplate reader (Bio-Rad). All experiments were repeated three times.

### Wound healing assay

The migration abilities of U87 and T98G cells were assessed via a wound-healing assay. When cells grown on the plate reached a density of approximately 90 %, an artificial wound was created by scraping the confluent monolayer with a 200-µL plastic pipette tip. Cell migration was monitored under a microscope at 0 and 24 h, and images were taken immediately. The average wound closure rate was measured in triplicate and the results were evaluated with ImageJ software (NIH, Bethesda, MD, USA).

### Cell migration and invasion assays

Cell migration and invasion were evaluated using 24-well transwell chambers (Corning, Inc., Corning, NY, USA) coated with Matrigel or not. Briefly, transfected U87 and T98G cells (2 × 10^4^) were seeded into the upper chambers containing serum-free medium, whereas medium containing 20 % fetal bovine serum was added to the lower chambers. After incubation for 24 h (migration assays) and 48 h (invasion assays) at 37 °C, the cells were fixed with 4 % formalin and stained with 0.1 % crystal violet. The cell invasion assay was performed in the same manner, but the upper chamber was covered with Matrigel. Cells were counted under a light microscope in randomly selected fields and analyzed using ImageJ software.

### Statistical analyses

Statistical analyses were conducted using R (version 3.6.1, The R Foundation, Vienna, Austria) and SPSS (version 24.0, SPSS, Inc., Chicago, IL, USA). Statistical analysis involved Student’s *t* -test, Mann-Whitney *U* test, analysis of variance, or two-way repeated measures analysis of variance with post-hoc Student’s *t*-test or paired Student’s *t*-test. Differences with p values < 0.05 were considered as statistically significant.

## Results

### Identification of DEGs and differentially expressed mRNAs (DEMs)

**Fig. 2:**
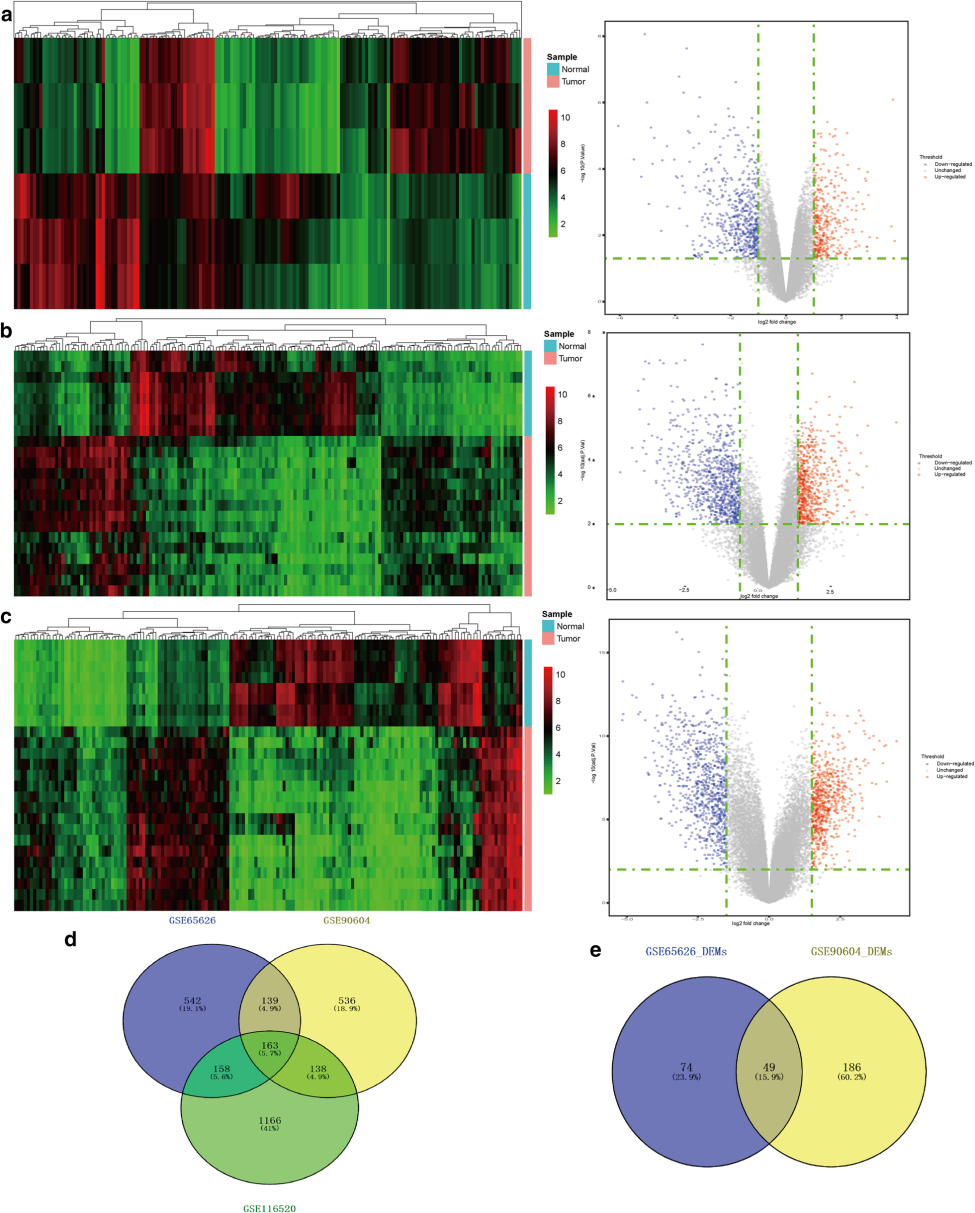
Expression profiles of DEGs and DEMs. **a–c** Heat map and volcano map of DEG expression levels in the **a** GSE65626 dataset, **b** GSE116520 dataset and **c** GSE116520 dataset. **d** Venn diagram showing DEGs in the three datasets. **e** Venn diagram showing DEMs in the two microRNA datasets. The red nodes represent upregulated DEGs with FDR < 0.01 and logFC > 1 or > 1.5; the green nodes represent downregulated DEGs with FDR < 0.01 and logFC < − 1.0 or < − 1.5. *DEGs* differentially expressed genes. *DEMs* differentially expressed microRNAs

We evaluated data from 35 patients with GBM and 18 healthy controls. GSE90604, GSE65626, and GSE116520 were analyzed using RStudio software. We identified 163 DEGs; 82 were upregulated and 81 were downregulated (Table 1; Fig. 2a–d). Among the DEMs, we identified 49 microRNAs, including 29 and 20 up- and downregulated miRNAs, respectively (Table 2; Fig. 2e). The top 10 upregulated genes were *TM1P1*, *COL4A1*, *TNC*, *CA12*, *PLAU*, *CKS2*, *TMEM45A*, *PLOD2*, *NCAPG*, and *SERPINH1*. The top 10 downregulated genes were *CAMK2A*, *OPALIN*, *AK5*, *GRIN1*, *SLC17A7*, *SH3GL3*, *MOBP*, *VSNL1*, *UNC13C*, and *SYN1*.

**Table 1:** 163 DEGs were identified from GSE65626, GSE90604, GSE116520 in the patients with GBM to healthy control

DEGs	Gene name
Up-regulated	*CLIC1,KDELR2,COL4A1,CKS2,TIMP1,VIM,LAMC1,NCAPG,CD44,FCGBP,LAMA4,PYGL,ANGPT2,PDPN,WEE1,TMEM45A,ETS1,CD93,SERPINH1,P4HA1,TGFB1I1,CALU,ANXA1,ABCC3,TSPAN6,IGF2BP3,GBP2,TNFRSF1A,GAS2L3,GBE1,CCNA2,TPX2,ARHGAP18,GADD45A,DLGAP5,ID3,EZH2,TNC,STEAP3,NAMPT,SLC43A3,IQGAP2,EMP3,CDH11,IFI30,PHLDA1,PLP2,KIF4A,FNDC3B,COL4A2,ADAMTS9,FSTL1,CD163,VEGFA,PRRX1,NES,LMNB1,PLOD2,ANO6,EMP1,FAM20C,SMC4,TNFRSF19,IGFBP5,ITGA5,GBP1,PTGFRN,CALD1,CDCA7L,CFI,ABCA1,HAS2,HIST1H2BK,FAM129A,MIR21,SPRY1,ZNF217,C1S,PCDHB16,KLHDC8A,CRISPLD1,DCBLD2*
Down-regulated	*PEX5L,RAB11FIP4,NECAB1,UNC13C,DLG2,OPALIN,MAST3,AK5,CACNG3,PCLO,CAMK2A,GRIN1,SYN2,KIAA0513,SLC17A7,RIMS2,GRM3,PPP1R16B,LGI3,TMEM130,CA11,RAPGEF4,SEC14L5,CREG2,NRGN,MAP7D2,NEFM,GNAO1,SYN1,SYT1,SH3GL3,HHATL,SV2B,KIAA1107,SCN2B,PPP2R2C,CNTNAP2,ANKS1B,MAP7,GABRA5,CNTNAP4,MAL,MOBP,RUNDC3A,SLCO1A2,PPFIA2,PTPRD,NPTX1,ATP2B2,NTSR2,SNAP25,ZNF536,KLHL32,GABRA2,EPB41L4B,RAPGEF5,ATP1B1,CAMKV,DBC1,FA2H,PLLP,NKAIN2,SNAP91,CLDN10,ENPP2,SNCA,SERPINI1,CACNA2D3,KIF1A,MOG,TMEM144,MAG,KLK6,GNG3,CNTN2,PTGDS,ERMN,CNDP1,TF,HSPA2,RCAN2*

*FDR < 0.01 and |Foldchange(FC)|>1

**Table 2:** 21 DEMs were identified from GSE65626, GSE116520 in the patients with GBM to healthy control

DEMs	Gene Name
Up-regulated	*hsa-miR-21-5p,hsa-miR-106b-5p,hsa-miR-210-3p,hsa-miR-18b-5p,hsa-miR-21-3p,hsa-miR-10b-5p,hsa-miR-424-3p,hsa-miR-23a-3p,hsa-miR-195-5p,hsa-miR-371b-5p,hsa-miR-27a-3p,hsa-miR-8063,hsa-miR-5001-5p,hsa-miR-195-3p,hsa-miR-130a-3p,hsa-miR-214-3p,hsa-miR-24-2-5p,hsa-miR-6068,hsa-miR-629-5p,hsa-miR-106b-3p*
Down-regulated	*hsa-miR-138-2-3p,hsa-miR-874-3p,hsa-miR-874-5p,hsa-miR-139-3p,hsa-miR-184,hsa-miR-338-5p,hsa-miR-330-5p,hsa-miR-770-5p,hsa-miR-323a-3p,hsa-miR-29c-3p,hsa-miR-487b-3p,hsa-miR-6743-5p,hsa-miR-3200-3p,hsa-mir-139,hsa-miR-383-5p,hsa-miR-758-5p,hsa-miR-128-3p,hsa-miR-433-3p,hsa-miR-769-5p,hsa-miR-139-5p,hsa-miR-129-5p,hsa-miR-330-3p,hsa-miR-1250-5p,hsa-miR-338-3p,hsa-mir-770,hsa-miR-29b-3p,hsa-miR-138-5p,hsa-miR-485-5p,hsa-miR-769-3p*

*FDR < 0.01 and |Foldchange(FC)|>1

### Functional and signal pathway enrichment analysis

**Fig. 3:**
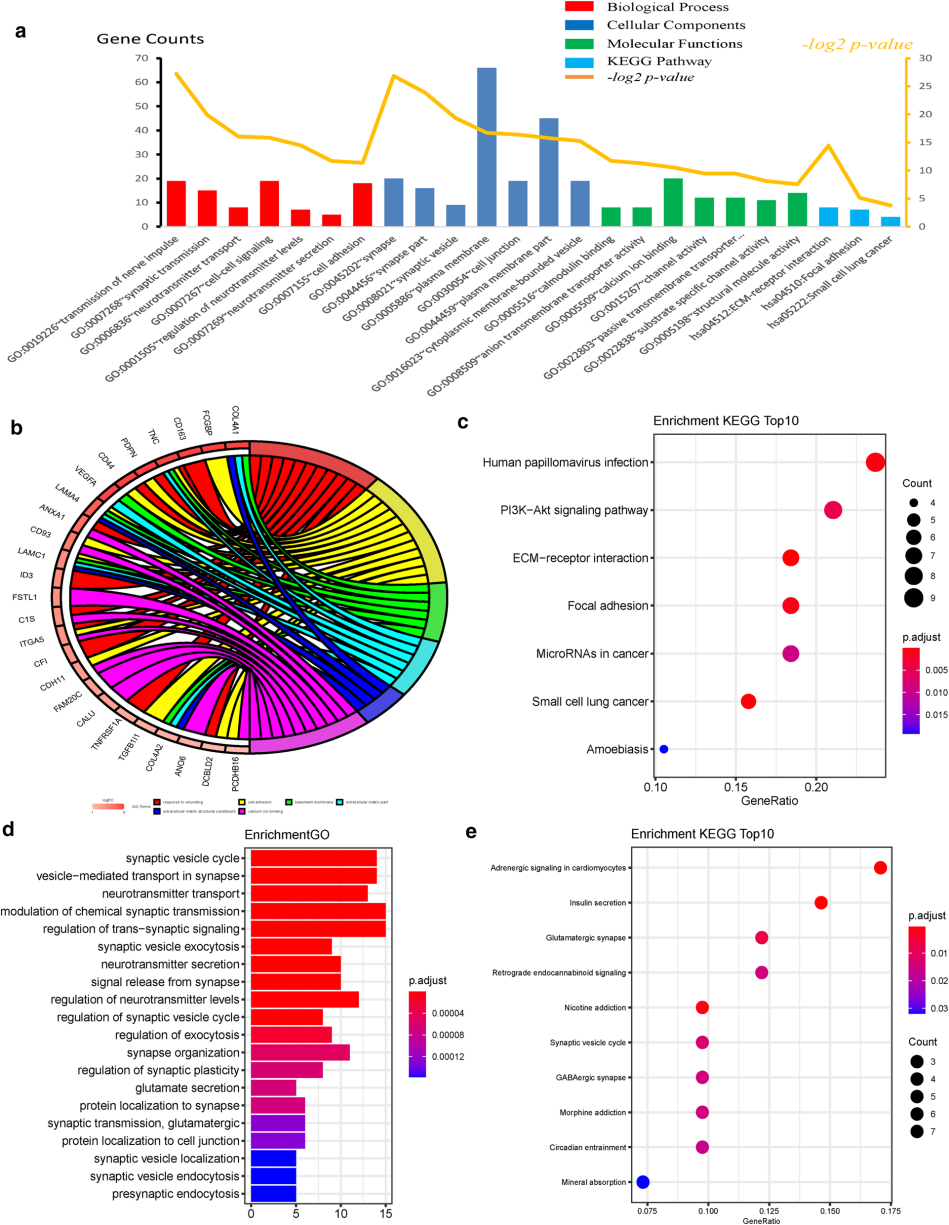
Gene Ontology and Kyoto Encyclopedia of Genes and Genomes enrichment analysis of DEGs**.** GO enrichment analysis of DEGs. **a** DEGs were classified as BP, CC, or MF. **b**, **c** Ranking of significantly enriched GO terms for upregulated DEGs. **d**, **e** Ranking of significantly enriched GO terms for downregulated DEGs. *GO* Gene Ontology, *BP* biological process, *CC* cellular component, *MF* molecular function, *DEGs* differentially expressed genes

We uploaded the selected DEGs to the online website DAVID to identify the enriched GO terms and KEGG pathways. The DEGs were classified as BP, CC, or MF (Fig. 3a). The first two significant functions were selected for analysis (Fig. 3b). As shown in Table 3, GO analysis showed that the DEGs were most significantly enriched in the transmission of nerve impulses. Moreover, upregulated DEGs were significantly enriched in the extracellular matrix (ECM) receptor interaction pathway and basement membrane, and particularly in protein binding (Fig. 3c). Downregulated DEGs were enriched in functions such as transmission of nerve impulse, synapse, and synapse part (Fig. 3d). The results of KEGG pathway analysis indicated enrichment in adrenergic signaling in cardiomyocytes (Fig. 3e).

**Table 3:** The miRCODE, miRTarbase, and TargetScan database revealed interactions ceRNA network

miRNA	mRNA	lncRNA
*hsa-miR-29b-3p*	*SERPINH1*	*H19 C2orf48 FAM87B C22orf34 HCP5 POLR2J4 LINC00475 UBAC2-AS1 TPRG1-AS1 GAS5 LINC00461 SNHG10 MIR210HG PVT1*
*has-miR-29c-3p*	*SERPINH1*	*H19 C2orf48 FAM87B C22orf34 HCP5 POLR2J4 LINC00475 UBAC2-AS1 TPRG1-AS1 GAS5 LINC00461 SNHG10 MIR210HG PVT1*
*hsa-miR-139-5p*	*DCBLD2*	*LINC00324 HCP5 LINC00152 GAS5 SNHG3 LINC00461 PVT1*
*hsa-miR-129-5p*	*C1S*	*LINC00324 C22orf34 SNHG12 LINC00466 ZNF503-AS1 HCG15 HOTAIR C9orf147 DLEU2 HOTAIRM1 MIR155HG SNHG3 WNT5A-AS1 CRNDE MIR210HG HAS2-AS1 PCAT1 SNHG1*

### PPI analysis and gene module analysis

**Fig. 4:**
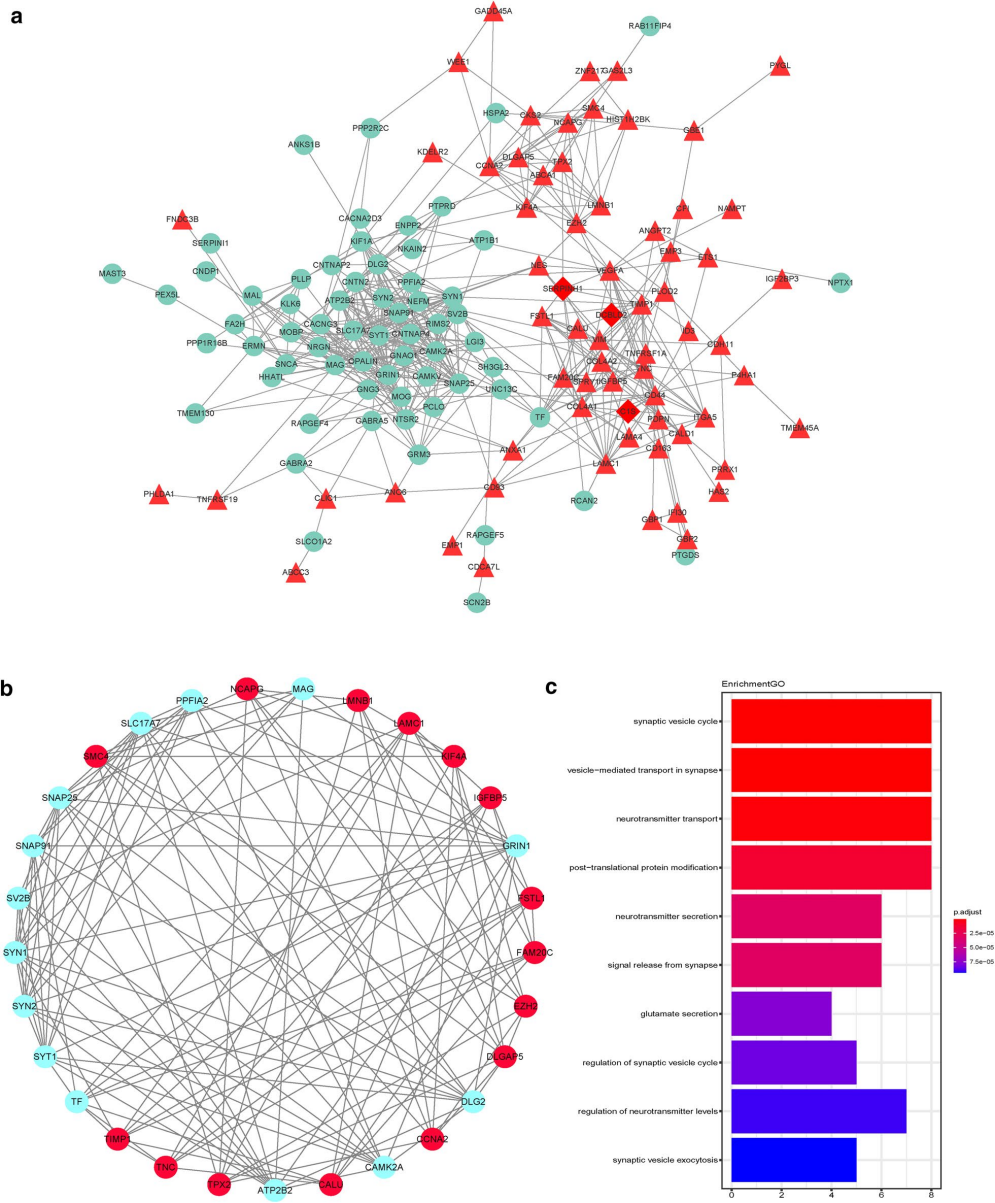
PPI network of DEGs. Node color: red indicates upregulated genes, sky-blue indicates downregulated genes. **a** PPI network based on the STRING online database contained 132 nodes and 484 edges. **b** Most significant module identified by MCODE (score = 9.071). **c **GO enrichment analysis of modules. *PPI* protein-protein interaction, *DEGs* differentially expressed genes, *GO* Gene Ontology

A total of 132 DEGs was screened using the STRING online database and Cytoscape software. Sixty-seven upregulated genes and sixty-five downregulated genes were included in the DEG PPI network (Fig. 4a); 30 genes were excluded from the PPI network (132 nodes and 484 edges). After filtering with a cutoff of node degree ≥ 6, the top 10 hub genes were *SYN1*, *SYT1*, *SNAP25*, *SYN2*, *SLC17A7*, *GRIN1*, *ATP2B2*, *DLG2*, *CAMK2A*, and *SNAP91.* Using Cytoscape’s MCODE plug-in, module 1 (score = 9.071), consisting of 29 nodes and 127 edges (Fig. 4b), was selected as an important module in the PPI network. In the functional module, the functions of important genes were annotated and analyzed. Functional enrichment analysis showed that module genes were mainly involved in the synaptic vesicle cycle (Fig. 4c).

### Prognostic significance of DEGs

**Fig. 5:**
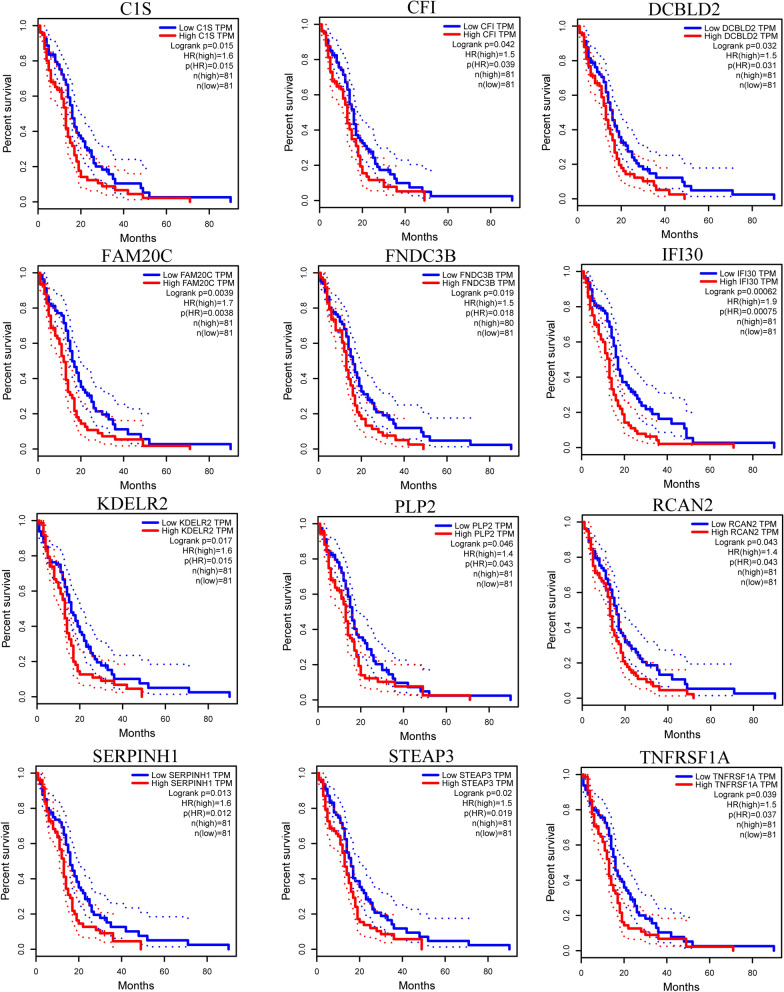
Kaplan–Meier curve of 12 DEGs significantly correlated with overall survival. *DEGs* differentially expressed genes

The GEPIA database was used to analyze the overall survival (OS) rates of patients with GBM at 20, 40, 60, and 80 months, as well as the correlation between the DEGs and survival prognosis of patients with GBM. The results showed that 12 DEGs (*C1S*, *CFI*, *DCBLD2*, *FAM20C*, *FNDC3B*, *IFI30*, *KDELR2*, *RCAN2*, *PLP2*, *SERPINH1*, *STEAP3*, and *TNFRSF1A*) were significantly correlated with the OS rate of patients with GBM (p < 0.05) (Fig. 5).

### Construction of ceRNA network

**Fig. 6:**
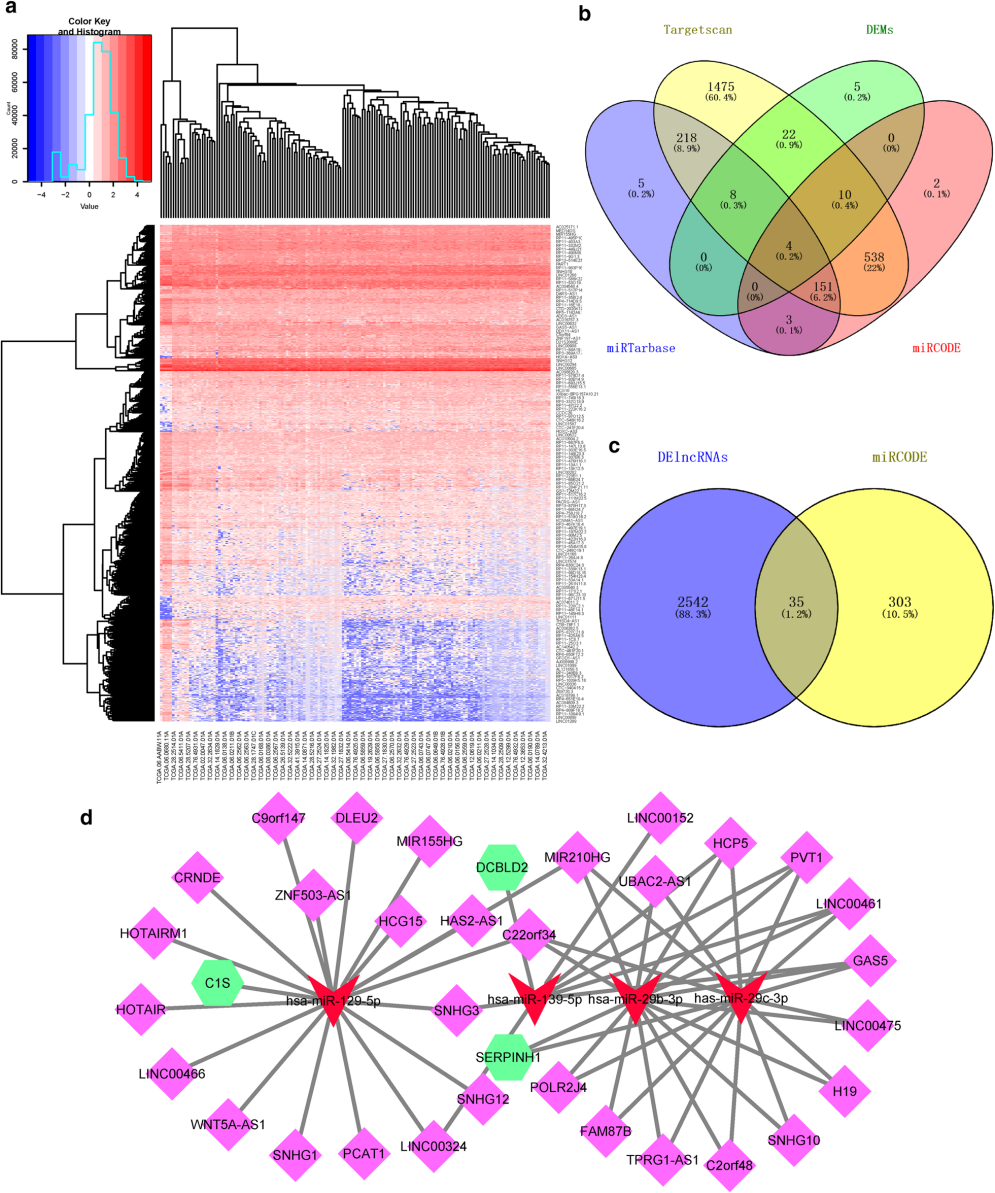
LncRNA-miRNA-mRNA ceRNA network analysis. **a** DElncRNAs from TCGA. DE lncRNAs with FDR-adjusted *p* < 0.01 and logFC > 1. **b** Four DEMs conformed to the miRcode, miRTarBase, and TargetScan databases according to the Venn diagram. **c** Venn diagrams showing 35 overlapping lncRNAs were screened out between the 2577 DE lncRNAs in TCGA and 338 lncRNAs predicted by miRcode, which could bind to four DEMs. **d** Red color represents miRNA downregulation, fluorescent green polygons represent mRNA upregulation, and light-purple diamonds represent lncRNA upregulation. *DElncRNAs* differentially expressed lncRNAs, *TCGA* The Cancer Genome Atlas, *DEMs* differentially expressed mRNAs

We identified 2577 DE lncRNAs in TCGA dataset using the edgeR package (Fig. 6a). miRcode, miRTarBase, and TargetScan were used to predict the miRNA-mRNA pairs. Only miRNA-mRNA pairs aligned across the three databases were incorporated into the ceRNA network. Four DEMs met the requirements of these databases (Fig. 6b). Thirty-five overlapping lncRNAs were screened out from the 2577 DE lncRNAs in TCGA and 338 LncRNAs that could bind to four DEMs and were predicted by miRcode. (Fig. 6c). According to the ceRNA hypothesis, lncRNA can act as a ceRNA to enhance the expression of target genes by sponging miRNAs. Finally, Cytoscape (Version 3.7.1) was used to verify the initial ceRNA network (Fig. 6d and Table 3).

**Fig. 7:**
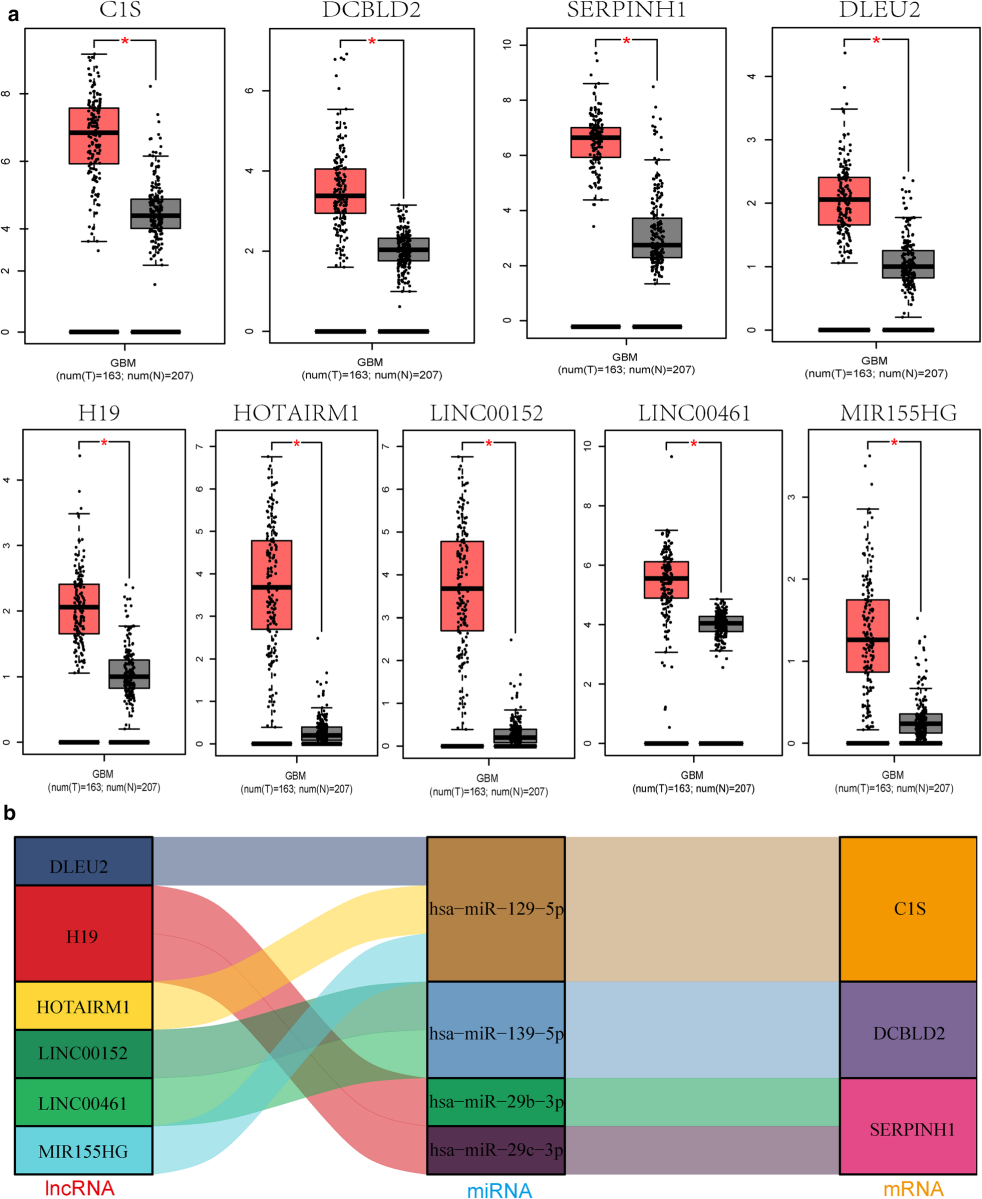
Expression of genes and construction of the GBM lncRNA-miRNA-mRNA network. **a** Expression of nine key genes, including 6 lncRNAs and 3 mRNAs, in GBM and normal tissue samples from the GEPIA databases (*p < 0.05). **b** Ji mulberry figure revealing four pairs of ceRNA networks: H19/miR-29b-3p/SERPINH1, H19/miR-29c-3p/SERPINH1, LINC00152 LINC00461/miR-139-5p/DCBLD2, and MIR155HG HOTAIRM1 DLEU2/ miR-129-5p/C1S. *GBM* glioblastoma, *GEPIA* Gene Expression Profiling Interactive Analysis

To evaluate the DE lncRNAs in the ceRNA network, we screened highly expressed DE lncRNAs from the boxplots in the GEPIA module. We next validated the potential DE lncRNAs targeted by four DEMs using the lncBace2.0 database. Third, we verified the expression of *DLEU2*, *H19*, *HOTAIRM1*, *LINC00152*, *LINC00461*, *MIR155HG*, *C1S*, *DCBLD2*, and *SERPINH1* in GBM tumor tissues and normal brain tissues using TCGA database. *DLEU2*, *H19*, *HOTAIRM1*, *LINC00152*, *LINC00461*, and *MIR155HG* were identified as DE lncRNAs (p < 0.05), whereas *C1S*, *DCBLD2*, and *SERPINH1* were identified as DE mRNAs (p < 0.05) (Fig. 7a). The final ceRNA network was used to construct regulatory axes and included 6 lncRNAs, 4 miRNAs, and 3 mRNAs (Fig. 7b and Table 4).

**Table 4:** Four lncRNA-miRNA-mRNA networks

lncRNA	miRNA	mRNA
*H19*	*has-miR-29b-3p*	*SERPINH1*
*H19*	*hsa-miR-29c-3p*	*SERPINH1*
*LINC00152 LINC00461*	*has-miR-139-5p*	*DCBLD2*
*MIR155HG HOTAIRM1 DLEU2*	*hsa-miR-129-5p*	*C1S*

### Validation of potential ceRNA axis and correlation analysis of triads

**Fig. 8:**
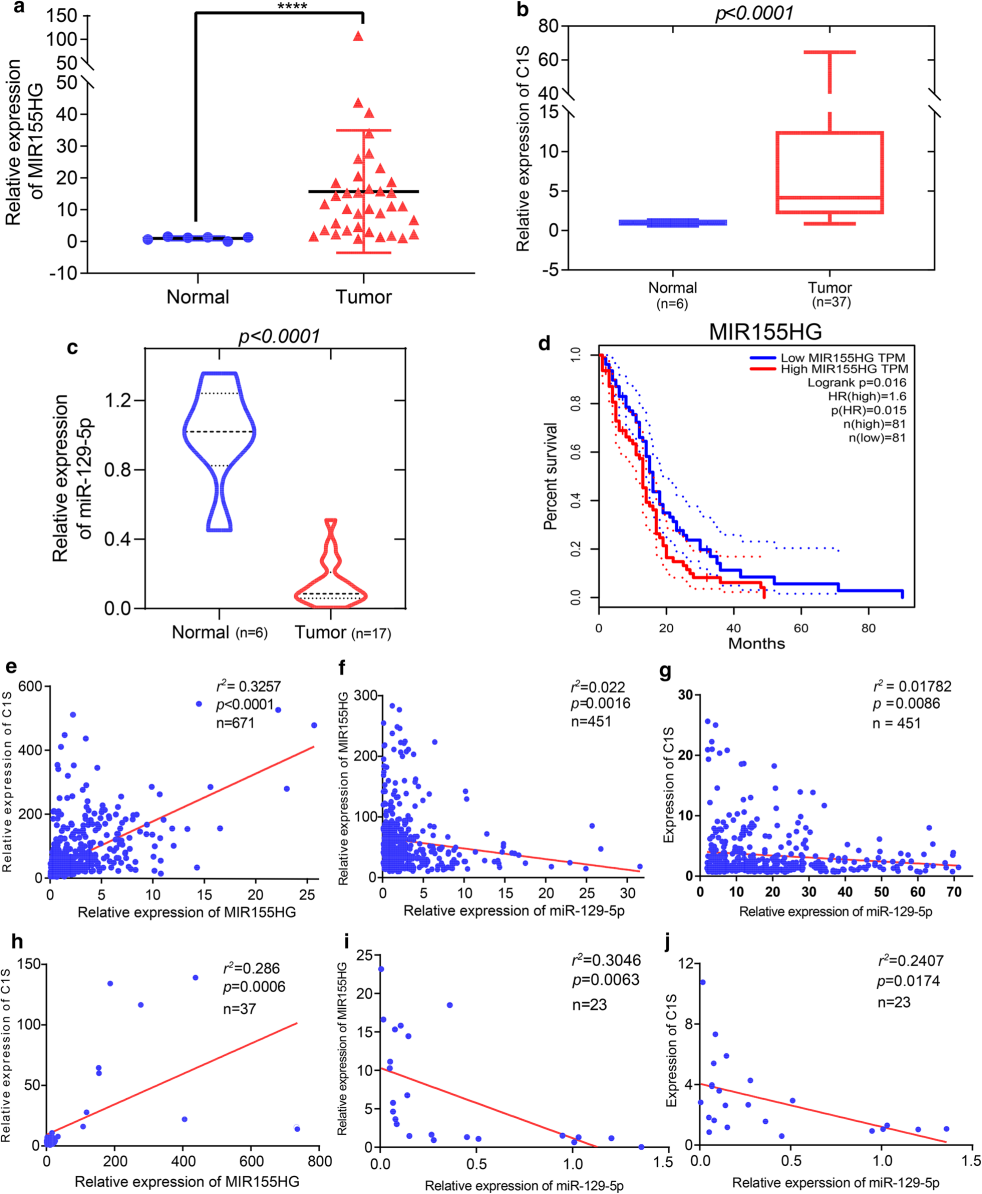
Expression and correlation of the MIR155HG/ miR-129-5p/C1S axis in GBM. **a** Expression of MIR155HG was significantly increased in gliomas compared to in normal brain tissues according to qRT-PCR analysis. **b** Expression of C1S in GBM (n = 37) and normal brain tissue samples collected at our institution. **c** Expression of miR-129-5p was significantly reduced in gliomas compared to in normal brain tissues according to qRT-PCR analysis. **d **High level of MIR155HG expression was significantly associated with the overall survival of patients with glioma in TCGA dataset. **e**, **g **Correlation between MIR155HG/C1S mRNA, miR-129-5p/MIR155HG, and miR-129-5p/C1S mRNA expression in TCGA dataset was analyzed by Pearson test. **h**, **j **Correlation between MIR155HG/C1S mRNA, miR-129-5p/MIR155HG, and miR-129-5p/C1S mRNA expression was analyzed by Pearson test and qRT-PCR. *GBM* glioblastoma, *qRT-PCR* real-time quantitative reverse-transcription polymerase chain reaction, *TCGA* The Cancer Genome Atlas. ****p < 0.0001

*MIR155HG/miR-129-5p/C1S* was identified as a potential regulatory axis from the ceRNA network for the following reasons: the results of qRT-PCR analysis indicated that *MIR155HG* (Fig. 8a) and *C1S* (Fig. 8b) were significantly upregulated in GBM (n = 37), whereas *miR-129-5p* (Fig. 8c) was significantly downregulated compared to in normal brain tissues. The results of OS analysis of *MIR155HG* indicated that *MIR155HG* had prognostic value in GBM (Fig. 8d). The glioma RNA sequencing data were downloaded from TCGA and converted into transcripts per million, which was verified by the results of qRT-PCR and Pearson coefficient correlation. The expression of *MIR155HG* and *C1S* were positively correlated (Fig. 8e, h). In contrast, the expression of *MiR-129-5p* and *MIR155HG* (Fig. 8f, i), as well as *miR-129-5p* and *C1S* (Fig. 8 g, j) were negatively correlated. Therefore, *MIR155HG/miR-129-5p/C1S* may be an important ceRNA axis.

### Knockdown of C1S inhibited the proliferation, migration, and invasion abilities of GBM cells

**Fig. 9:**
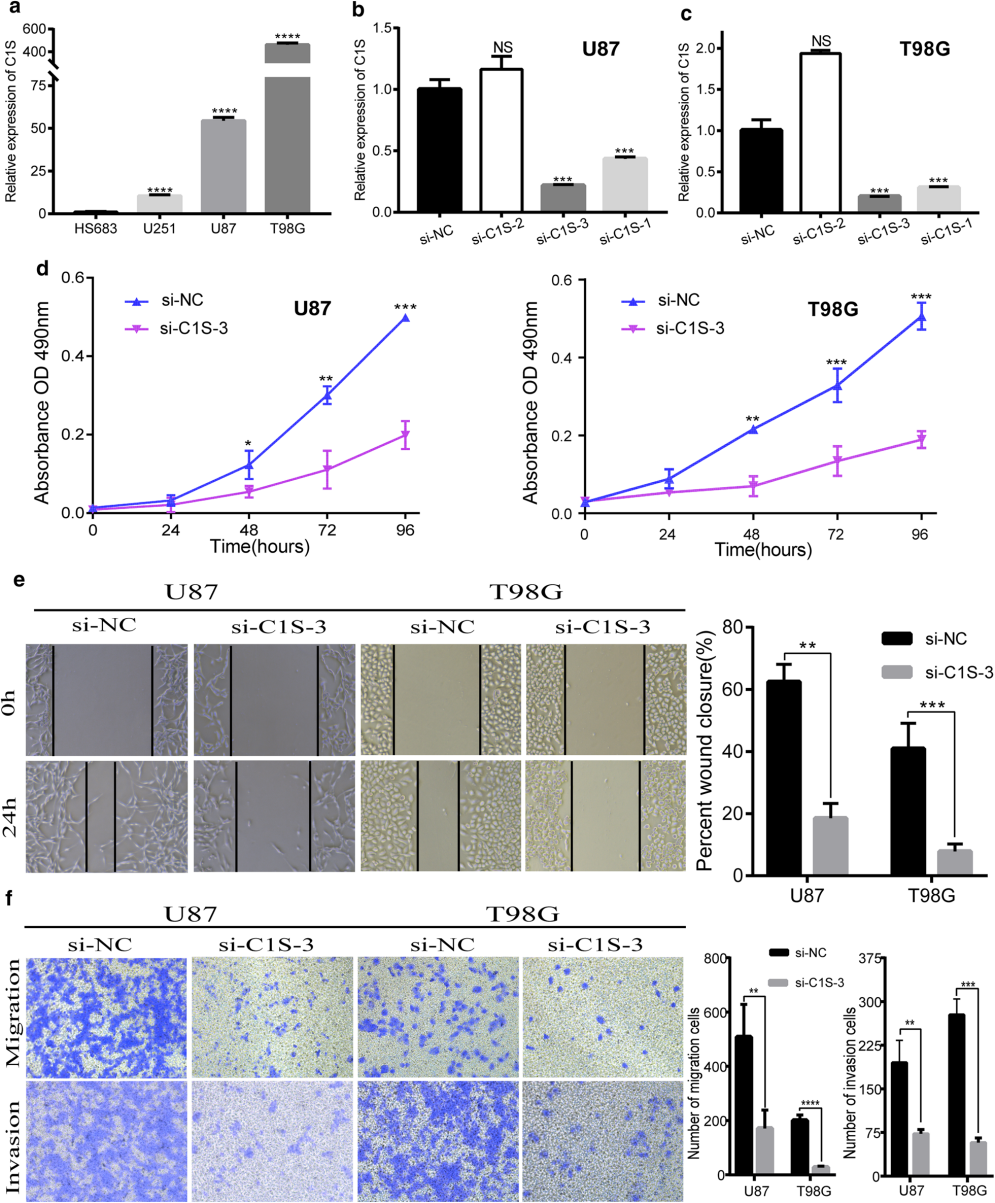
Depletion of C1S suppressed the proliferation, migration, and invasion abilities of glioblastoma cell lines. **a **Expression of C1S expression in glioblastoma cell lines. **b**, **c **Expression of C1S was downregulated in the siRNA group in U87 and T98G cells. **d **Suppression of the proliferation of U87 and T98G cells transfected with si-C1S was detected by MTS assay. **e**, **f **Wound-healing assay and transwell assay revealed that downregulation of C1S significantly inhibited the invasion and migration of U87 and T98G cell lines compared with the NC groups. *p < 0.05; **p < 0.01; ***p < 0.001, ****p < 0.0001

To investigate the function of *C1S* in GBM, we evaluated the impact of *C1S* on GBM cell proliferation, migration, and invasion. First, *C1S* was detected in four GBM cell lines (HS683, U251, U87, T98G), with U87 and T98G cells showing high *C1S* expression (Fig. 9a). Next, three specific siRNAs were used to silence *C1S* expression in the U87 and T98G cell lines. After transfection and incubation for 48 h, evaluation of the interference efficiency of the siRNAs by RT-qPCR in U87 and T98G cells revealed that siRNA-*C1S*-3 showed the highest silencing efficiency (Fig. 9b, c). Compared with the NC group, the MTS assay showed that *C1S* knockdown significantly reduced the viability of U87 and T98G cells (Fig. 9d). To further explore the functions of *C1S* in the migration and invasion of GBM cells, a wound-healing assay was performed; *C1S* knockdown in U87 and T98G cells significantly inhibited the migration abilities of GBM cells at 24 h of incubation (Fig. 9e). Additionally, transwell assays showed that *C1S* knockdown decreased the migration and invasion abilities of U87 and T98G cells (Fig. 9f).

## Discussion

Despite the significant progress made in comprehensive therapy, the five-year prognosis of patients with glioma remains poor[19, 20]. In recent years, the ceRNA hypothesis has gained attention, with studies focusing on how imbalanced expression of ceRNA affects the pathogenicity and progression of cancer[21–23]. In the present study, we used expression spectrum data from three mRNA expression profiling datasets to identify intersecting DEGs in the datasets to ensure the accuracy of our results. Twelve DEGs associated with survival prognosis were selected to construct a ceRNA network, which was validated by qRT-PCR. This network provides insights into the involvement of ceRNA in the diagnosis and mechanism of glioma, as well as demonstrates the utility of using the ceRNA axis as a GBM biomarker.

A Venn diagram was constructed to further filter and refine the identified DEMs, DE miRNAs, and DE lncRNAs. Through GO and PPI network analysis, we found that the DE mRNAs were mainly enriched in cell adhesion and ECM-receptor interaction. For example, the interaction between integrins and the ECM promotes chemoresistance by protecting cells from drug-induced apoptosis in several cancer types, including small lung cancer, myeloma, and esophageal squamous-cell carcinoma[24, 25]. The migration and invasion of glioma cells involve a complex combination of multiple molecular processes, including those associated with the ECM, protease secretion, and the actin cytoskeleton, which alter the modification of tumor cell adhesion[25].

In general, lncRNAs can act as miRNA sponges to regulate the expression of downstream coding genes. MiRNAs often adsorb adjacent lncRNAs and mRNAs. First, three databases (miRcode, miRTarBase, and TargetScan) were used to analyze whether the DEGs and DEMs contained binding sites. Second, four selected DEMs were used to reverse-predict the potential binding sites of the lncRNAs using two databases (miRcode and lncBace2.0). Initially, we aimed to build a ceRNA network with 163 DEGs screened from the GEO mRNA microarray datasets. However, when we intersected the DEMs-DEGs, those with too many and non-critical genes were predominant. Unexpectedly, among the 163 DEGs, 12 were related to prognosis and survival. Therefore, we isolated and analyzed these DEGs. Finally, a ceRNA network map was constructed.

The 12 DEGs associated with OS were *C1S*, *CFI*, *DCBLD2*, *FAM20C*, *FNDC3B*, *IFI30*, *KDELR2*, *RCAN2*, *PLP2*, *SERPINH1*, S*TEAP3*, and *TNFRSF1A*. Among them, *DCBLD2*[26], *FNDC3B*[27], *PLP2*[28], *SERPINH1*[29, 30], *STEAP1*[31, 32], and *TNFRSF1A*[33] were found to be abnormally expressed in a series of human tumors. *IFI30* is the most stable prognostic gene among interferon-stimulated genes in glioma[34]. *CFI* is a potential therapeutic target for nonmelanoma skin cancer[35]. *C1S*, *FAM20C*, *KDELR2*, and *RCAN2* have rarely been reported in cancer. After screening three databases, miRcode, miRTarBase and TargetScan, we finally identified three mRNAs for constructing ceRNA networks, namely, *C1S*, *SERPINH1*, and *DCBLD2*.

The Ji mulberry figure illustrated the four established ceRNA axes: *H19/has-miR-29b-3p/ SERPINH1*, *DLEU2/miR-29c-3p/SERPINH1*, *LINC00152 LINC00461/miR-139-5p/DCBLD2*, and *MIR155HG HOTAIRM1 H19/miR-129-5p/C1S. MIR155HG* is a promising biomarker for tracking tumor progression and predicting metastasis[36] and should be further investigated in longitudinal studies of patients with early-stage tumors. Additionally, *MIR155HG*, which can also competitively bind to *miR-129-5p*, is an important regulatory factor for hematopoietic, inflammatory, immune, and tumor development processes, as well as other physiological and pathological processes[37]. Phosphorylated *DCBLD2* can recruit *TRAF6* and stimulate the AKT pathway, promoting the migration and invasion abilities of glioma cells[26]. *SERPINH1* expression is related to the malignancy of glioma and promotes angiogenesis in glioma through autocrine and paracrine mechanisms[38]. *C1S*, as a protease involved in the classical complement pathway[39], was recently reported to be associated with tumor immunity and the promotion of tumor growth[40]; however, it has not been studied as an oncogene in gliomas. We identified *C1S* as a new potential biomarker in glioma tissue based on its prognostic and OS-predictive value and predicted that *C1S* plays a crucial role in the ceRNA network. Analysis of the function of *C1S* revealed that downregulation of *C1S* contributed to the inhibition of proliferation, migration, and invasion of glioblastoma cell lines. Our research improves the understanding of the biological function of *C1S* in tumors and provides a basis for the further exploration of these aspects.

Although we established three network relationship chains of ceRNAs and verified a new ceRNA axis, providing insights into the role of ceRNA in glioma, there were a few limitations to this study. First, verification of the ceRNA axis based only on the correlation of the differential expression of genes is insufficient; further cell function experiments are needed to confirm our results. Second, three network chains of ceRNAs and only one ceRNA axis, which have not been reported previously, were selected for verification. Third, we only performed in vitro cell experiments to analyze the functions of *C1S*; further functional experiments should be performed in vivo to validate the in vitro findings.

In conclusion, *C1S* was found to be associated with a worse prognosis of GBM. Additionally, *C1S* knockdown inhibited the proliferation, invasion, and migration of GBM cell lines. Thus, *C1S* may serve as a new biomarker for GBM. Our findings provide insight into the roles of ceRNAs in GBM.

## Supplementary Information

**Additional file 1. **In vitro experiment

## Data Availability

The datasets used and analyzed during the current study are available from the corresponding author on reasonable request. The TCGA and GEO datasets could be obtained from the online website.

## Abbreviations

TCGA: The Cancer Genome Atlas
DEMs: Differentially expressed mRNAs
GBM: Glioblastoma
qRT-PCR: Real-time quantitative reverse-transcription polymerase chain reaction
GEPIA: Gene Expression Profiling Interactive Analysis
