# Correction to: Construction of a competitive endogenous RNA network and analysis of potential regulatory axis targets in glioblastoma

**DOI:** 10.1186/s12935-023-03035-0

**Published:** 2023-08-29

**Authors:** Kai Yu, Huan Yang, Qiao-li Lv, Li-chong Wang, Zi-long Tan, Zhe Zhang, Yu-long Ji, Qian-xia Lin, Jun-jun Chen, Wei He, Zhen Chen, Xiao-li Shen

**Affiliations:** 1 Department of Neurosurgery, The Second Afliated Hospital of Nanchang University, No. 1 Minde Road, Donghu District, Jiangxi, 330006 Nanchang People’s Republic of China; 2 Jiangxi Key Laboratory of Translational Cancer Research, Jiangxi Cancer Hospital, Jiangxi, Nanchang People’s Republic of China; 3 Jiangxi University of Traditional Chinese Medicine, Jiangxi, Nanchang People’s Republic of China

**Correction: Cancer Cell Int (2021) 21:102**

10.1186/s12935-021-01789-z

Following the publication of the original article [1], we were notified of an error in Fig. 7. The corrected Fig. 7 can be found below.

**Fig. 7:**
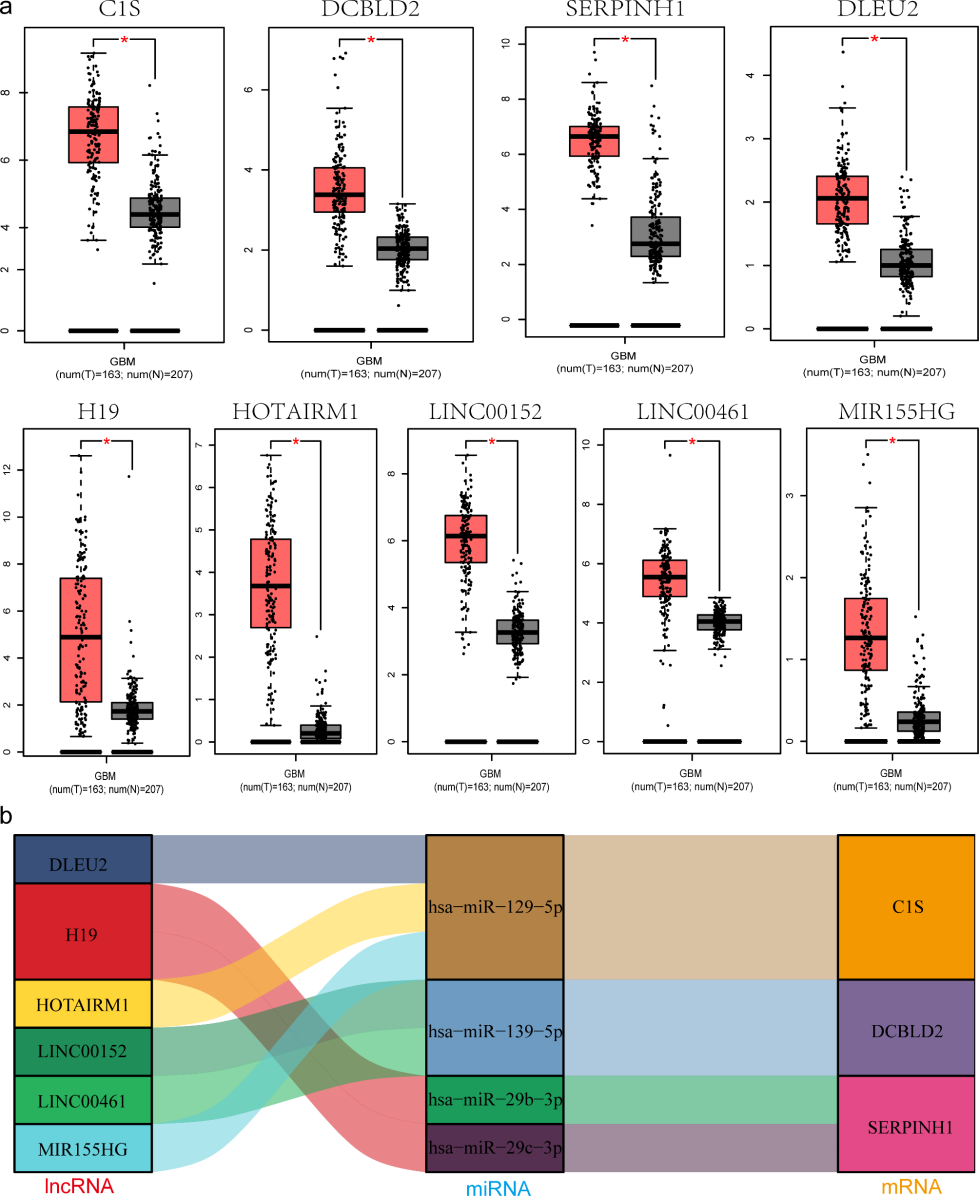
Expression of genes and construction of the GBM lncRNA-miRNA-mRNA network. **a** Expression of nine key genes, including 6 lncRNAs and 3 mRNAs, in GBM and normal tissue samples from the GEPIA databases (*p < 0.05). **b** Ji mulberry figure revealing four pairs of ceRNA networks: H19/miR-29b-3p/SERPINH1, H19/miR-29c-3p/SERPINH1, LINC00152 LINC00461/miR-139-5p/DCBLD2, and MIR155HG HOTAIRM1 DLEU2/ miR-129-5p/C1S. GBM glioblastoma, GEPIA Gene Expression Profiling Interactive Analysis
